# Supplementary material for: Does Diabetes Mellitus Increase the Risk of Avascular Osteonecrosis? A Systematic Review and Meta-Analysis
Source: Int J Environ Res Public Health. 2022 Nov 18;19(22):15219. doi: 10.3390/ijerph192215219 (PMC9690760; doi:10.3390/ijerph192215219)
Supplement: Supplementary file 1 [file ijerph-19-15219-s001.zip › Supplementary File S2. Systematic Literature Review Protocol.pdf]

Systematic Literature Review Protocol

Does diabetes mellitus increase the risk of avascular osteonecrosis? A systematic review and meta-analysis

Date of Protocol: 29.07.2022
Version number: 1.0

Glossary

"Literature" throughout this protocol refers to: scientific original articles; reports (including short reports), data retrieved from clinical trial database; data presented at congresses.

Rationale

The pathogenesis of non-traumatic osteonecrosis is unclear, but there are several established risk factors such as corticosteroid treatment, alcohol abuse, rheumatic diseases, bone-marrow transplantation, or antiretroviral treatment. Diabetes mellitus (DM), which causes disease of both large vessels (macroangiopathy) and small vessels (microangiopathy), can also increase the risk of avascular osteonecrosis (AVN). The evidence on the association between diabetes and the risk of AVN in sites other than the jaw is insufficient.

Objectives

The main objective of the literature review is to determine the association between DM and the risk of AVN.

Methodology

This protocol was developed in accordance with the Preferred Reporting Items for Systematic review and Meta-Analysis Protocols (PRISMA-P) criteria [4].

Information sources

The PubMed and EMBASE databases will be searched until 29th July 2022 in English, Polish, or Spanish. Relevant keywords will be applied alone or in combinations for identification of the data. The search strategy alongside the number of hits is presented in the Supplementary Table S4.

Eligibility criteria

Eligibility criteria for this review are summarized in Table S1. Literature search questions were constructed according to the PICOS approach: Population, Intervention, Comparator, Outcomes, and Study Design. These criteria will be used to determine the eligibility of each study for inclusion in the review.

Table S1. Eligibility criteria for studies.

	Inclusion criteria	Exclusion criteria
**Population**	• Adults aged ≥ 18 years<br>• Patients with AVN located in other place than jaw	• Patients without AVN<br>• Patients with jaw AVN
**Intervention**	• Not applicable	• Not applicable
**Comparator**	• Patients with DM vs without DM	• No comparator
**Outcomes**	• The risk of AVN	• Outcomes of interest not reported
**Study design**	• Cohort studies<br>• Case-control studies<br>• Cross-sectional studies<br>• Registry/database studies<br>• Randomized controlled trials	• Case reports, case series<br>• Reviews, editorials, letters, comments, notes<br>• Animal or *in vitro* studies<br>• Pharmacokinetic studies<br>• Cost-effectiveness studies
**Language restrictions**	• Studies published in English, Spanish and Polish	• Studies published in languages other than English, Spanish and Polish
**Publication date**	• Studies published until 29.07.2022	• Studies published after 29.07.2022

Data management

Bibliography and abstracts for all citations identified through the PubMed and EMBASE search will be exported into Endnote version X9 to facilitate removal of duplicate citations and screening of titles and abstracts.

Selection process

1. Titles and abstracts identified by the PubMed search will be screened by two independent reviewers to assess potential eligibility for inclusion, using the eligibility criteria presented in **Error! Reference source not found.**. Reasons for exclusion will be briefly documented.

2. For abstracts potentially meeting the inclusion criteria, full text publications will be retrieved. Each study will be assessed for eligibility by two independent reviewers, according to the criteria presented in **Error! Reference source not found.**. Reasons for exclusion will be briefly documented.

3. Outcomes of the selection process, including the number of studies identified in the search, the number excluded at each stage and reasons for exclusion, will be documented in the form of a PRISMA flow diagram.

Data collection process

Data will be extracted in a consistent manner from studies meeting the eligibility criteria. Extraction will be performed by two independent reviewers and all extracted data will be collated within a table in Microsoft Excel.

Data items

The proposed list of variables that will be extracted from eligible studies is presented in Table. The reviewer will note where the given data item is "not reported" or "not applicable".

Table S2. Proposed data items to be extracted from eligible studies.

Item	Description
**Bibliographic details**	
Author	Author(s) surname(s)
Year	Year of publication
Title	Article title
Publication type	Full text or Abstract
**Study characteristics**	
Study design	Type of study
Cohort size	Total number of patients included in the study. If applicable, also the number of patients receiving intervention and comparator
Characteristics of AVN case	
Corticosteroid use	Total number of patients using corticosteroids
**Baseline population characteristics**	
Baseline age	Mean (SD) age of study patients at baseline
Sex	Number (%) of male and female patients
**Study outcomes – Medical benefit**	
Risk of AVN	Odds ratios (OR) of AVN.

Articles not meeting inclusion criteria will be excluded from further appraisal. The reasons for exclusion, apart from not meeting the above-mentioned inclusion criteria, include some specific exclusion criteria related to the study methodology, type of publication, as well as detection of duplicates. During this initial selection, a code will be attributed to each excluded

article (Table S3). It is possible that some articles will have more than one reason to be excluded (for example: article not in a retained language and outside of the scope), for which only one will be listed as the reason for exclusion.

Table S3. Example of application of the code for exclusion.

Code	Description	
NE	Non-Equivalence	Different treatment, different indication
M	Methodology	No comparator, no statistical analysis, inappropriate clinical outcome
T	Type of study	Any other type of study not mentioned in study design criteria
OS	Out of scope	Outside of the scope
L	Language	Foreign language not generally understood
D	Duplicate	Duplicate article (same author, same study...)

Outcomes and prioritisation

The main outcome of interest in this review is effect of DM on the risk of AVN.

Risk of bias in individual studies

Risk of bias in the individual studies included in this review will be assessed using the Newcastle-Ottawa Scale.

Data synthesis

We will calculate odds ratios (OR) using inverse variance for the risk of AVN in patients with diabetes vs. without diabetes (OR > 1 indicate an increased risk of AVN in patients with diabetes). A random-effects meta-analysis will be carried out with the restricted maximum-likelihood estimator for tau2 and the Q-profile method for the confidence interval of tau2 and tau. Heterogeneity will be expressed with the I2 and $\tau 2$ statistics, and it will be evaluated with Cochran's Q test. A prediction interval will be estimated to take heterogeneity into account. Sensitivity analyses will include leave-one-out analyses. Publication bias will be assessed using the Peters regression test and by inspection of a funnel plot. A P-value of less than 0.05 will be considered statistically significant. The R software (version 4.1.3) will be used for all analyses.

Supplementary Table S4. PubMed and EMBASE search strategy.

Search phrases:

**PubMed:**
Phrase 1: ((AVN) OR (avascular necrosis) OR (sterile necrosis) OR (ischemic necrosis) OR (osteonecrosis)) AND (diabet* OR hyperglycemia OR (glucose)) AND ("risk factor" OR risk OR "OR")

Phrase 2: ((AVN) OR (avascular necrosis) OR (sterile necrosis) OR (ischemic necrosis) OR (osteonecrosis)) AND (diabet* OR hyperglycemia OR (glucose)) AND ("risk factor" OR risk)
**EMBASE:**
('diabetes mellitus'/exp OR 'diabetes' OR 'diabetes mellitus' OR 'diabetic') AND (('risk factor'/exp OR 'relative risk' OR 'risk factor' OR 'risk factors') OR ('risk assessment'/exp OR 'risk adjustment' OR 'risk analysis' OR 'risk assessment' OR 'risk evaluation') OR ('high risk population'/exp OR 'high risk group' OR 'high risk population' OR 'risk group') OR ('odds ratio'/exp OR 'OR (odds ratio)' OR 'odds ratio')) AND ('avascular necrosis'/exp OR 'avascular bone necrosis' OR 'avascular hip necrosis' OR 'avascular necrosis' OR 'avascular osteonecrosis' OR 'bone infarction' OR 'bone necrosis, avascular' OR 'coagulation bone necrosis' OR 'coagulation osteonecrosis' OR 'coagulative bone necrosis' OR 'coagulative osteonecrosis' OR 'ischaemic bone necrosis' OR 'ischaemic osteonecrosis' OR 'ischemic bone necrosis' OR 'ischemic osteonecrosis' OR 'necrosis, avascular' OR 'osteonecrosis, avascular')
